# Accelerating Oxygen Electrocatalysis Kinetics on Metal–Organic Frameworks via Bond Length Optimization

**DOI:** 10.1007/s40820-024-01382-9

**Published:** 2024-04-19

**Authors:** Fan He, Yingnan Liu, Xiaoxuan Yang, Yaqi Chen, Cheng-Chieh Yang, Chung-Li Dong, Qinggang He, Bin Yang, Zhongjian Li, Yongbo Kuang, Lecheng Lei, Liming Dai, Yang Hou

**Affiliations:** 1 Key Laboratory of Biomass Chemical Engineering of Ministry of Education, College of Chemical and Biological Engineering, Zhejiang University, Hangzhou, 310027 People’s Republic of China; 2 Institute of Zhejiang University - Quzhou, Quzhou, 324000 People’s Republic of China; 3 Ningbo Institute of Materials Technology and Engineering, Chinese Academy of Sciences, Ningbo, 315201 People’s Republic of China; 4 School of Biological and Chemical Engineering, NingboTech University, Ningbo, 315100 People’s Republic of China; 5 Department of Physics, Tamkang University, New Taipei, 25137 Taiwan, People’s Republic of China; 6 Australian Carbon Materials Centre (A-CMC), School of Chemical Engineering, University of New South Wales, Sydney, NSW 2051 Australia

**Keywords:** Metal–organic frameworks, Bond length adjustment, Spin state transition, Orbitals hybridization, Water splitting

## Abstract

Metal–organic frameworks (MOFs) have been developed as an ideal platform for exploration of the relationship between intrinsic structure and catalytic activity, but the limited catalytic activity and stability has hampered their practical use in water splitting. Herein, we develop a bond length adjustment strategy for optimizing naphthalene-based MOFs that synthesized by acid etching Co-naphthalenedicarboxylic acid-based MOFs (donated as AE-CoNDA) to serve as efficient catalyst for water splitting. AE-CoNDA exhibits a low overpotential of 260 mV to reach 10 mA cm^−2^ and a small Tafel slope of 62 mV dec^−1^ with excellent stability over 100 h. After integrated AE-CoNDA onto BiVO_4_, photocurrent density of 4.3 mA cm^−2^ is achieved at 1.23 V. Experimental investigations demonstrate that the stretched Co–O bond length was found to optimize the orbitals hybridization of Co 3*d* and O 2*p*, which accounts for the fast kinetics and high activity. Theoretical calculations reveal that the stretched Co–O bond length strengthens the adsorption of oxygen-contained intermediates at the Co active sites for highly efficient water splitting.

**Supplementary Information:**

The online version contains supplementary material available at 10.1007/s40820-024-01382-9.

## Highlights

- The acid etching Co-naphthalenedicarboxylic acid-based metal–organic frameworks (donated as AE-CoNDA) catalyst displayed an excellent oxygen evolution reaction (OER) activity for long-term stability.
- Integration of the AE-CoNDA cocatalyst into BiVO_4_ achieved a remarkable PEC-OER activity.
- The stretched Co-O bond length regulated the spin state transition at the Co active sites.
- The optimized high spin state of Co sites adjusted the orbitals hybridization of Co 3*d* and O 2*p*.

**Supplementary Information:**

The online version contains supplementary material available at 10.1007/s40820-024-01382-9.

## Graphical abstract

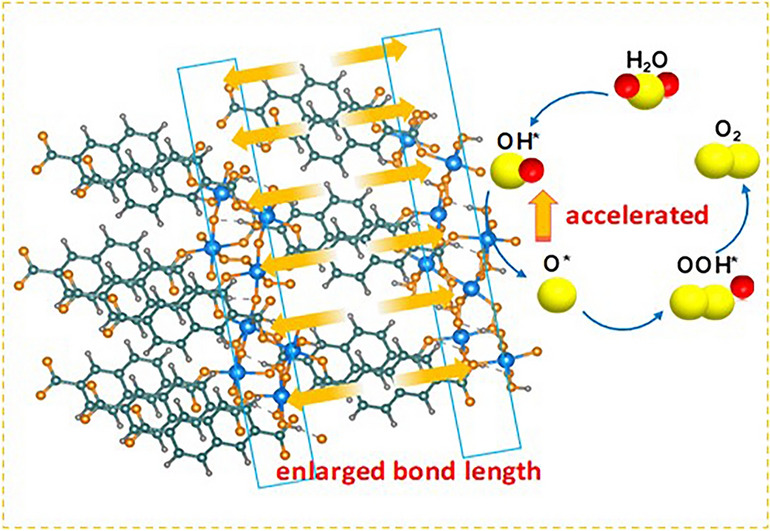

## Introduction

Developing efficient and durable cocatalysts is regarded as one of the most significant efforts in promoting photoelectrochemical (PEC) water splitting [1–5]. Compared with the cathodic hydrogen evolution reaction (HER) [6], the sluggish reaction kinetics [7, 8] and complicated four-electron transfer pathway [9–12] for the anodic oxygen evolution reaction (OER) remain a major bottleneck to limit their commercial uses [13–15]. While various strategies have been developed to accelerate sluggish reaction kinetics for the OER, its performance and stability still need to be compromised [16–18].

Recently, various oxygen evolution cocatalysts (OECs) [19–22] have been exploited to obtain desired PEC-OER performance [23]. Traditional OECs often exhibited ill-defined structures undesirable for mechanistic studies. Fortunately, metal–organic frameworks (MOFs) with well-defined crystal structures [24, 25] and abundant exposed active sites [26–28] are ideal for investigating the intrinsic structure and catalytic activity relationship [29–31]. Up to date, several researchers developed MOF materials decorated photoelectrode, such as MOF-BiVO_4_, to improve PEC-OER activity; however, their conductivity and catalytic stability are invariably unsatisfied. MOFs materials as precursors often need to be converted into carbon-based OECs via high-temperature calcination, thus causing severe structure collapse and metal agglomeration [32, 33]. Therefore, it is highly desirable to develop uncarbonized MOFs materials as active and durable OECs through rational modification [34].

For the OER processes in alkaline, it typically involves a four-electron transfer pathway and complicated deprotonation process of oxygen-contained intermediates (e.g., OH*, O*, and OOH*) [35]. Conventional MOFs materials usually contain saturated coordinated metal nodes that are unfavorable for adsorption of oxygen-contained intermediates. In addition, the framework structure of traditional MOFs is vulnerable to severe collapse under harsh condition. Thereby, it is important to modify the crystal structure of MOFs for boosting the OER activity and enhancing photoelectrode’s stability. Like the natural enzyme structure [24], distorted structure of catalyst has an effect on electronic spin configuration of metal sites, which is closely associated with the binding energy for oxygen-contained intermediates on the metal sites. Compared to thermal-induced or photoinduced method, acid etching is simplicity of operation and easily control distorted degree.

In particular, a bond length adjustment strategy was represented to regulate the intrinsic electronic structure of Co-2,6-naphthalenedicarboxylic acid-based MOFs (AE-CoNDA) catalyst by acid etching treatment. The AE-CoNDA catalyst showed a superior OER performance and rapid kinetics in alkaline media, featured by a low overpotential of 260 mV to reach 10 mA cm^−2^ and a small Tafel slope of 62 mV dec^−1^. Integration of the AE-CoNDA cocatalyst into a BiVO_4_ for solar water splitting led to an AM 1.5G photocurrent density of 4.30 mA cm^−2^ at 1.23 V, which outperformed the PEC-OER performance for most reported Co-based BiVO_4_ photoanodes (Table S1). Experimental results verified that the electronic structure on the Co active center in the AE-CoNDA was reconfigured by the bond length adjustment. An optimized bond length corresponded to the spin state transition from an intermediate spin (e_g_^1^t_2g_^6^) to a high spin (e_g_^2^t_2g_^5^) at the Co active site, facilitating the oxygen-contained intermediates adsorption. Theoretical calculations uncovered the optimized adsorption energy between the metal sites with optimal bond length and oxygen-contained intermediates for enhancing the OER performance.

## Experimental Procedures

### Synthesis of AE-CoNDA

2,6-naphthalenedicarboxylic acid (2,6-NDA, 172.9 mg) and CoCl_2_·6H_2_O (190.2 mg) were dissolved in a mixed solution containing 32-mL dimethyl formamide (DMF), 2-mL ethanol, and 2-mL deionized water. Thereafter, 1.0-mL triethylamine (TEA) was injected into the above solution mixture under vigorous stirring to form a uniform colloidal suspension. The formed suspension was sealed and further ultrasonicated for 300 min at 70 kHz and room temperature. The obtained sample was further immersed in a 0.2-mM acetic acid solution for acid etching. The resultant precipitate was washed with DMF and ethanol for three times, and dried in vacuum oven at 60 °C.

### Characterization

Morphologies of the as-prepared samples were obtained on a field emission scanning electron microscopy (FESEM) (Hitachi SU-8010), transmission electron microscopy (TEM) (HT7700), and high-resolution transmission electron microscopy (HR-TEM) (JEOL JEM-2001F). The crystal structures of the as-prepared samples were analyzed by X-ray powder diffraction (XRD) (Empyrean 200,895) using Cu Kα radiation. Chemical structures of the as-prepared samples were measured by X-ray photoelectron spectroscopy (XPS, Escalab250Xi) with Al Kα radiation. Raman spectra were obtained by a LabRAM HR Evolution unit. The metal content in samples was analyzed by inductively coupled plasma mass spectrometry (ICP-MS) (Vista Axial). The X-ray absorption spectroscopic (XAS) measurements of the as-prepared samples were conducted in Beijing Synchrotron Radiation Facility and Taiwan Synchrotron Radiation Facility. Quantum Design MPMS-7 superconducting quantum interference device (MPMS-VSM) magnetometer was utilized to obtain the magnetic properties of the as-prepared catalysts. The electron paramagnetic resonance (EPR) data of the as-prepared catalysts were acquired on a Bruker EMXmicro.

### Electrochemical Measurements

All electrochemical measurements were carried out by an electrochemical analyzer (CHI 760E) in a typical three-electrode cell. A saturated calomel electrode (SCE, CH Instruments) was used as the reference electrode, and a graphite rod was used as the counter electrode. The potential was converted to reversible hydrogen electrode (RHE) via a Nernst equation (*E*_RHE_ = *E*_SCE_ × 0.244 V + 0.0591 × pH). To evaluate the OER activities, the scan rate of linear sweep voltammetry (LSV) was set as 1.0 mV s^−1^ with the potentials from 1 to 1.8 V vs. RHE in 1.0 M KOH. Electrochemical impedance spectroscopy (EIS) was measured at 1.5 V vs. RHE with a frequency range from 10^5^ to 0.01 Hz. All polarization curves were calibrated with iR correction unless noted. Cyclic voltammetry cycles (CVs) at 0.96–1.06 V vs. RHE with the scan rates from 10 to 50 mV s^−1^ were applied.

### PEC-OER Measurements

The PEC-OER measurements were conducted with a front-side illumination in all cases (light enters from the absorber side). A saturated calomel electrode (SCE, CH Instruments) and a Pt wire were used as reference electrode and counter electrode, respectively. The recorded potential vs. SCE (*E*_SCE_) was converted against RHE using the Nernst equation (*E*_RHE_ = *E*_SCE_ × 0.244 V + 0.0591 × pH). A 1.0 M potassium borate buffer, prepared by adjusting the pH of 1.0 M H_3_BO_3_ to 9.0 with KOH solution, was used as the electrolyte. The stability of photoanode was evaluated by measuring curves of the potential vs. time at 1.0, 2.0, and 2.5 mA cm^−2^.

### Computational Methods

All spin-polarized density functional theory (DFT) calculations were performed with Vienna Ab initio Simulation Package (VASP). In this work, the generalized gradient approximation (GGA) within Perdew–Burke–Ernzerhof (PBE) method was used to describe the exchange–correlation interaction. The core electrons were replaced by the projector augmented wave (PAW) pseudopotential, and 450-eV plane-wave expansion was setup for energy cut-offs. The k-points sampling was set to 1 × 1 × 1 Monkhorst–Pack k-points grid for geometrical optimization and that of electronic property calculation was 1 × 1 × 2. The convergence threshold was set to 10^–5^ eV and 0.05 eV Å^−1^ for energy and force, respectively. Weak interaction was described by DFT-D3 method using empirical correction in Grimme’s scheme.

The (1 × 2) surface of bulk CoNDA (100) was chosen, which contains 16 Co atoms, 83 O atoms, 96 C atoms, and 64 H atoms. A vacuum slab of about 15 Å was maintained in the super-cell configuration that was large enough for the calculations. To simulate the tensile state of structure, tensile CoNDA structure was constructed by expanding the lattice parameters of the pristine CoNDA in three directions by 5%.

## Results and Discussion

### Solar-driven Water Oxidation Activity of AE-CoNDA@BiVO_4_

AE-CoNDA as cocatalyst was synthesized by ultrasonic exfoliation of a mixed solution of CoCl_2_·6H_2_O, 2,6-NDA, and DMF, followed by acid etching at a predetermined concentration of acetic acid. The AE-CoNDA@BiVO_4_ was assembled by spraying the AE-CoNDA ink on BiVO_4_, as illustrated in Fig. S1. Morphological images (Figs. S2–S5) exhibited that the AE-CoNDA and control samples uniformly coated on the nanoworm-like BiVO_4_ surface. Especially, the HR-TEM image of AE-CoNDA@BiVO_4_ (Fig. S2) exhibits a lattice fringe of 1.18 nm for the interlayer spacing distance of naphthalene-based MOF with transition metal nodes along the [100] direction, which assigned to AE-CoNDA. In addition, in XRD pattern (Fig. S6), the diffraction peak below 10° stands for crystalline MOF structure, indicating that the AE-CoNDA is highly crystallized. Raman spectra (Fig. S7) also show obvious characteristic peaks which assigned to naphthalene-based MOF. Above results confirmed that AE-CoNDA was highly crystallized and successfully deposited on BiVO_4_ photoelectrode.

**Fig. 1:**
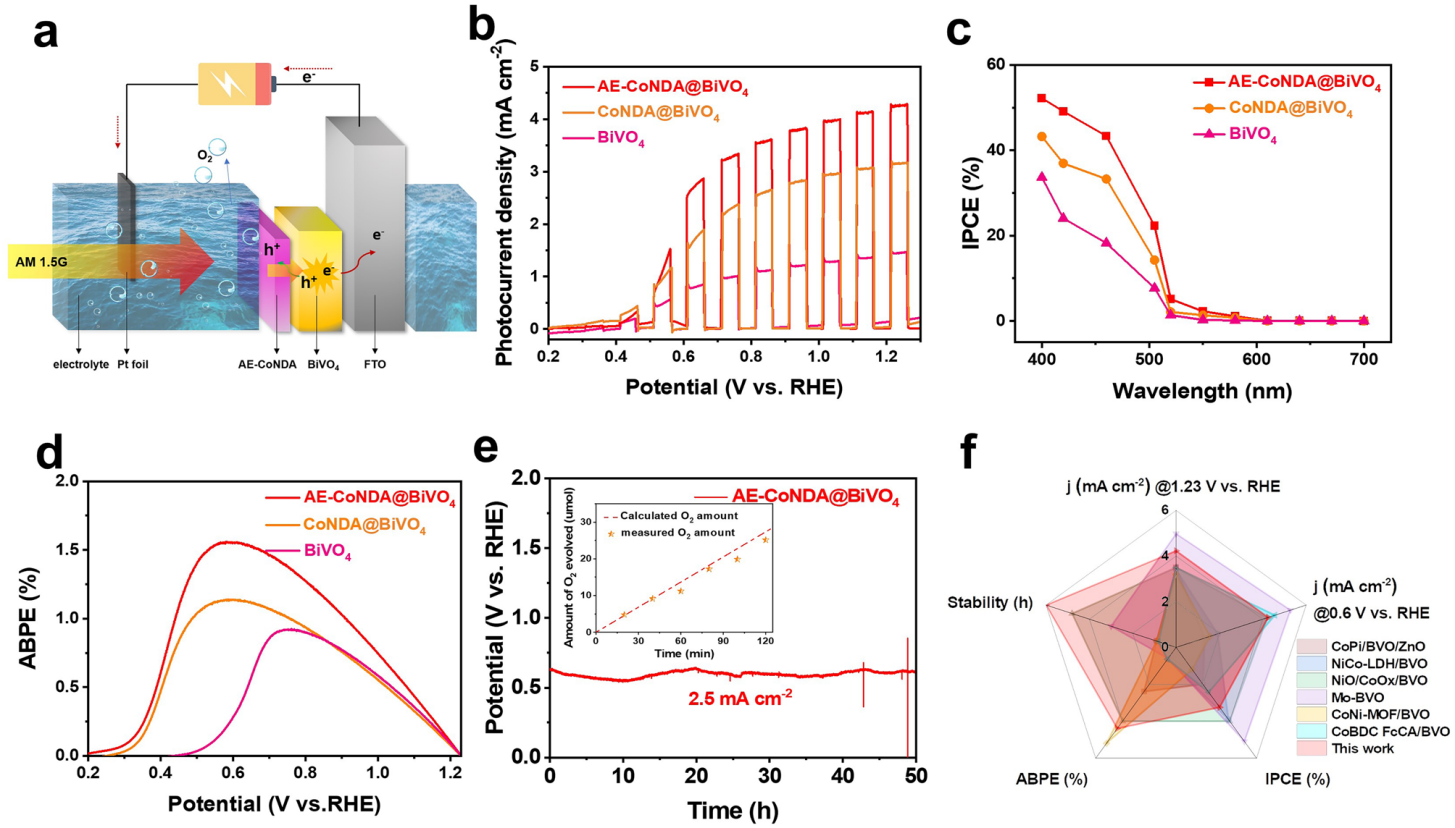
PEC-OER performances of AE-CoNDA@BiVO_4_. **a** Schematic diagram of working principle of a proposed PEC-OER cell. **b** Polarization curves of BiVO_4_, CoNDA@BiVO_4_, and AE-CoNDA@BiVO_4_ without adding sacrificial agent (Na_2_SO_3_) under chopped AM 1.5G irradiation. **c** IPCE curves and **d** ABPE curves of BiVO_4_, CoNDA@BiVO_4_, and AE-CoNDA@BiVO_4_ under AM 1.5G irradiation. **e** Stability of AE-CoNDA@BiVO_4_ under AM 1.5G irradiation at 2.5 mA cm^−2^, inset: amount of O_2_ evolution detected by gas chromatography and calculated from photocurrent during the PEC-OER of AE-CoNDA@BiVO_4_ at 0.6 V. **f** Photocurrent density at 0.6 V and 1.23 V, IPCE, ABPE, and stability of AE-CoNDA@BiVO_4_ compared with other reported Co-based BiVO_4_ photoanodes. All experiments were carried out in a 1.0 M potassium borate (pH = 9.0) solution

To demonstrate the catalytic performance, we first investigated PEC water oxidation property of AE-CoNDA@BiVO_4_ in alkaline electrolyte under AM 1.5G irradiation, as illustrated in Fig. 1a. The optimized AE-CoNDA@BiVO_4_ delivered a high photocurrent density of 4.30 mA cm^−2^ at 1.23 V (Figs. 1b and S8), which was much greater than that for BiVO_4_ alone (1.30 mA cm^−2^ at 1.23 V) and CoNDA@BiVO_4_ (3.20 mA cm^−2^ at 1.23 V). In addition, the AE-CoNDA@BiVO_4_ possessed a maximum incident photon-to-current conversion efficiency (IPCE) of 54.0% at 400 nm at 1.23 V (Fig. 1c), which was about 1.6 and 1.3 times higher than that of bare BiVO_4_ (33.0%) and CoNDA@BiVO_4_ (43.0%), respectively. AE-CoNDA@BiVO_4_ showed the maximum photon-to-current efficiency (ABPE) of 1.61% at 0.6 V (Fig. 1d), which was superior to that of BiVO_4_ (0.82% at 0.78 V) and CoNDA@BiVO_4_ (1.12% at 0.6 V). The lower charge transfer resistance for the AE-CoNDA@BiVO_4_ than that of CoNDA@BiVO_4_ and BiVO_4_ alone under light irradiation demonstrated that more effective interfacial charge transfer occurred at the AE-CoNDA@BiVO_4_ interface (Fig. S9). Moreover, clearly, it was seen that after introduction of AE-CoNDA, both charge transfer efficiency (η_transfer_) and charge transport efficiency (η_transport_) of AE-CoNDA@BiVO_4_ (Figs. S10–S13) increased throughout the whole potential range. The product analyses revealed that the generated photocurrent was mainly from the O_2_ production on AE-CoNDA@BiVO_4_ rather self-corrosion of photoelectrode (Fig. 1e). Besides, the potential on AE-CoNDA@BiVO_4_ was not raised for at least 50 h to maintain photocurrent density at 2.5 mA cm^−2^ (Fig. 1e), which is much durable than CoNDA@BiVO_4_ under 1.5 mA cm^−2^ and BiVO_4_ under 1.0 mA cm^−2^ (Fig. S14). Notably, the observed PEC-OER activity for AE-CoNDA@BiVO_4_ was regarded as an extreme high record among all reported Co-based BiVO_4_ hybrid photoanodes (Fig. 1f and Table S1). To further support the enhancement of photo-generated charge carrier transfer efficiency of BiVO_4_ after integrating AE-CoNDA cocatalyst, operando-irradiated XPS measurements [35, 36] were conducted (Fig. S15). The results revealed that the holes transferred from BiVO_4_ to AE-CoNDA cocatalyst occurred.

### Vital Role of AE-CoNDA as Cocatalyst

**Fig. 2:**
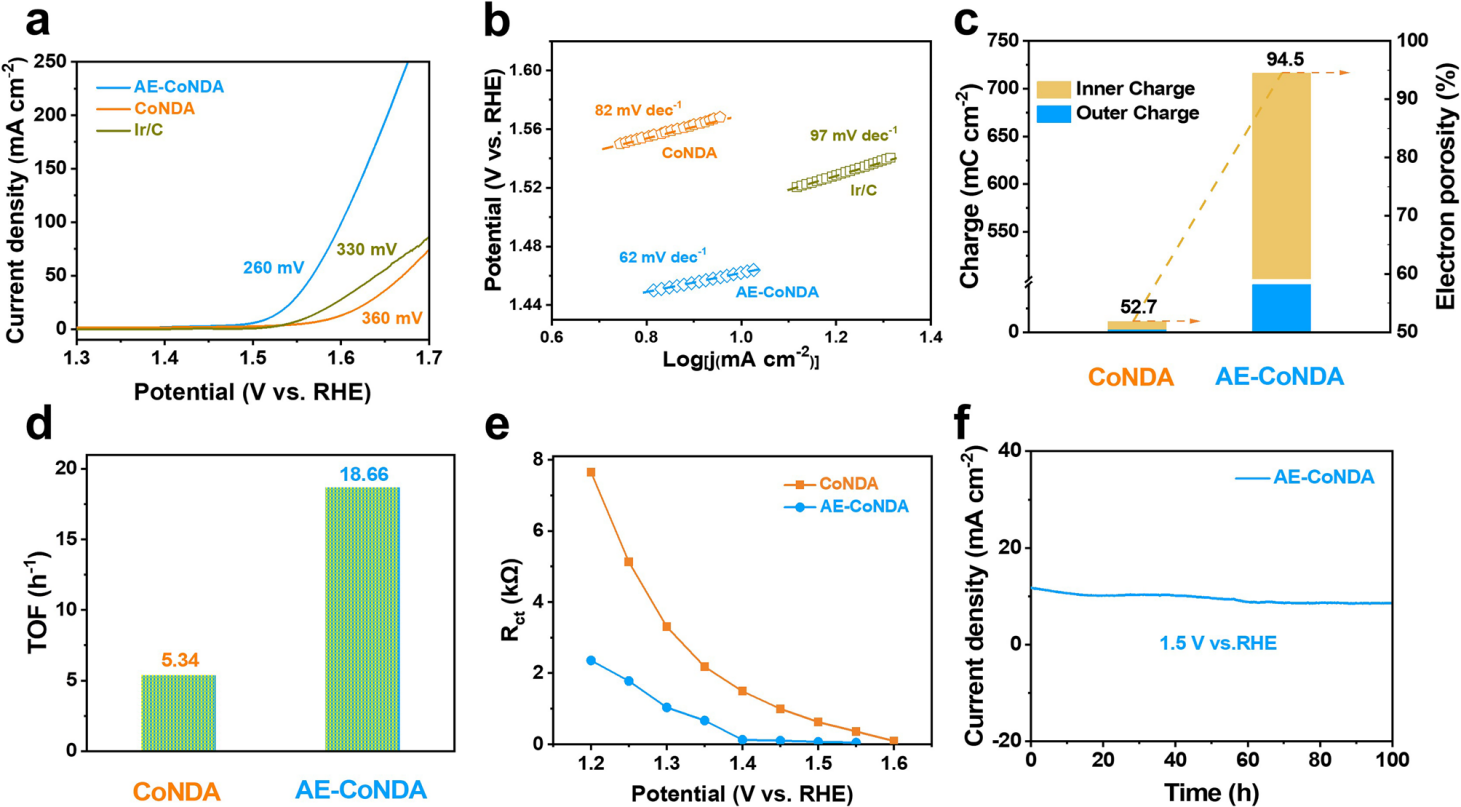
Electrocatalytic OER performances of AE-CoNDA.** a** Polarization curves and **b** Tafel slopes of AE-CoNDA, CoNDA, and Ir/C. **c** Internal and external voltammetric charge density and electron porosity of CoNDA and AE-CoNDA. **d** TOF values and **e** response of *R*_ct_ at different potentials for CoNDA and AE-CoNDA. **f** Chronopotentiometric durability of AE-CoNDA at 1.5 V

To probe the crucial role of AE-CoNDA as cocatalyst, the OER performance of AE-CoNDA was further evaluated in a three-electrode cell in 1.0 M KOH solution. The control samples with different reaction conditions were also synthesized to investigate the contribution of acetic acid etching. The optimal etching time was found to be 1.0 h, and optimal concentration of acetic acid was 0.2 mM (Figs. S16–S18). As shown in Fig. 2a, the as-prepared AE-CoNDA delivered only a low overpotential of 260 mV to reach a current density of 10 mA cm^−2^. Compared to the CoNDA without acid etching (360 mV), this result highlights the significant role of acid etching in enhancing the OER performance. Notably, the overpotential for the AE-CoNDA was even smaller than that of the benchmark Ir/C (330 mV) at the same current density, demonstrating the excellent OER activity for the former. The fast reaction kinetics of AE-CoNDA was further underscored by its smaller Tafel slope (62 mV dec^−1^) than that of CoNDA with 82 mV dec^−1^, and even commercial Ir/C with 97 mV dec^−1^, respectively (Fig. 2b). The AE-CoNDA further exhibited a much smaller electrochemical resistance (*R*_ct_) (Fig. S19 and Table S2) than that of the CoNDA, implying a fast charge transfer for AE-CoNDA. The ratio of inner charge to outer charge density for AE-CoNDA was calculated to be 94.5 (Figs. 2c and S20), which was much higher than that of CoNDA (52.7), as a result of the high electrochemical porosity. These results suggested that the acid etching not only increased the electrode roughness [37], but also produced abundant exposed active sites. The larger electrochemically active surface area (ECSA) of AE-CoNDA than that of CoNDA indicated the more electroactive surfaces for AE-CoNDA (Figs. S21 and S22). Moreover, the turnover frequency (TOF) of AE-CoNDA was calculated to be 18.66 h^−1^ at an overpotential of 0.3 V, which was approximately three times higher than the corresponding value for CoNDA (5.86 h^−1^) (Fig. 2d). Above results claimed that the increase in active surface area is not the main reason for the increase in the current density of the catalyst, but the intrinsic characteristics of the catalyst promote the catalytic activity of the catalyst, resulting in a higher current density.

To further validate the rapid charge transfer for AE-CoNDA, in situ EIS measurements were conducted (Table S2). The results showed that the *R*_ct_ value of AE-CoNDA was much lower than that of CoNDA over a wide range of potential (Fig. 2e), indicating the fast charge transfer efficiency for AE-CoNDA. Besides, the *R*_ct_ value of the AE-CoNDA became stable at low potential (1.4 V), demonstrated that the OER process was triggered at low potentials on the AE-CoNDA [38, 39]. Notably, the excellent alkaline OER performance in terms of the overpotential (260 mV at 10 mA cm^−2^) and Tafel slope (62 mV dec^−1^) for the AE-CoNDA was superior to those of almost all non-carbonized Co MOF-based OER electrocatalysts previously reported (Table S3). Moreover, the OER process on the AE-CoNDA was stable, no apparent change in the overpotential was observed over 100 h of continuous operation (Fig. 2f), which is much stable than CoNDA under same potential (Fig. S23). Especially, the XRD pattern (Fig. S24a) and Raman spectra (Fig. S24b) of AE-CoNDA after OER process remained similar curves with pristine AE-CoNDA, indicating structural stability of catalyst.

### Analysis of Intrinsic Structure on AE-CoNDA

**Fig. 3:**
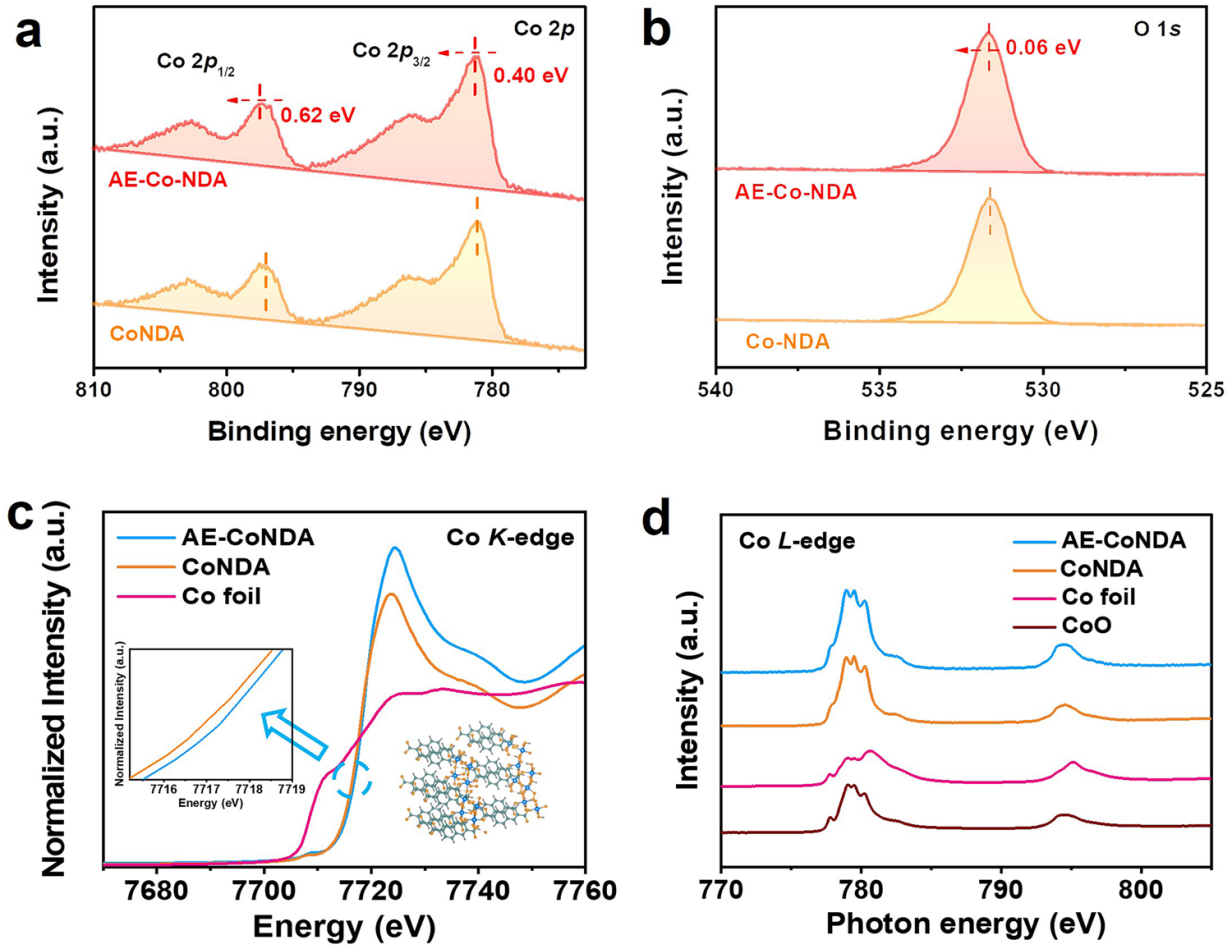
Electronic structure analysis of AE-CoNDA. High-resolution **a** Co 2*p* and **b** O 1*s* spectra of CoNDA and AE-CoNDA. **c** Co K-edge XANES spectra of AE-CoNDA, CoNDA, and Co foil. **d** Co L-edge XANES spectra of AE-CoNDA, CoNDA, CoO, and Co foil

To gain the insights of superior OER activity on AE-CoNDA, we first performed XPS measurements. In the high-resolution Co 2*p*, O 1*s*, and C 1*s* XPS spectra (Figs. 3a, b and S25), the Co 2*p* XPS peaks in the AE-CoNDA displayed a higher binding energy, shifted approxiately 0.62 and 0.40 eV, while O 1*s* XPS peaks positively shifted 0.06 eV to higher binding energy, as the peaks of O-C = O and C–C/C = C negatively shifted to lower binding energies (Fig. S25). These results demonstrated the electrons from Co atoms within AE-CoNDA transferred to the O atoms adjacent to C atoms and ultimately located on the C atoms. To deeply investigate the fine structure of AE-CoNDA, we further conducted X-ray absorption spectroscopy (XAS). The obtained X-ray absorption near-edge structure (XANES) spectra (Fig. 3c) showed the absorption edge with an increased photon energy of Co species within the AE-CoNDA in comparison with CoNDA and Co foil, indicating a higher valence state for Co species in AE-CoNDA. Moreover, the Co L-edge XAS spectra of the AE-CoNDA (Fig. 3d) exhibited a Co absorption edge shifted to a higher photon energy relative to that of the Co foil and CoNDA, but close to that of reference CoO, suggesting the average Co valence state of + 2 for AE-CoNDA.

**Fig. 4:**
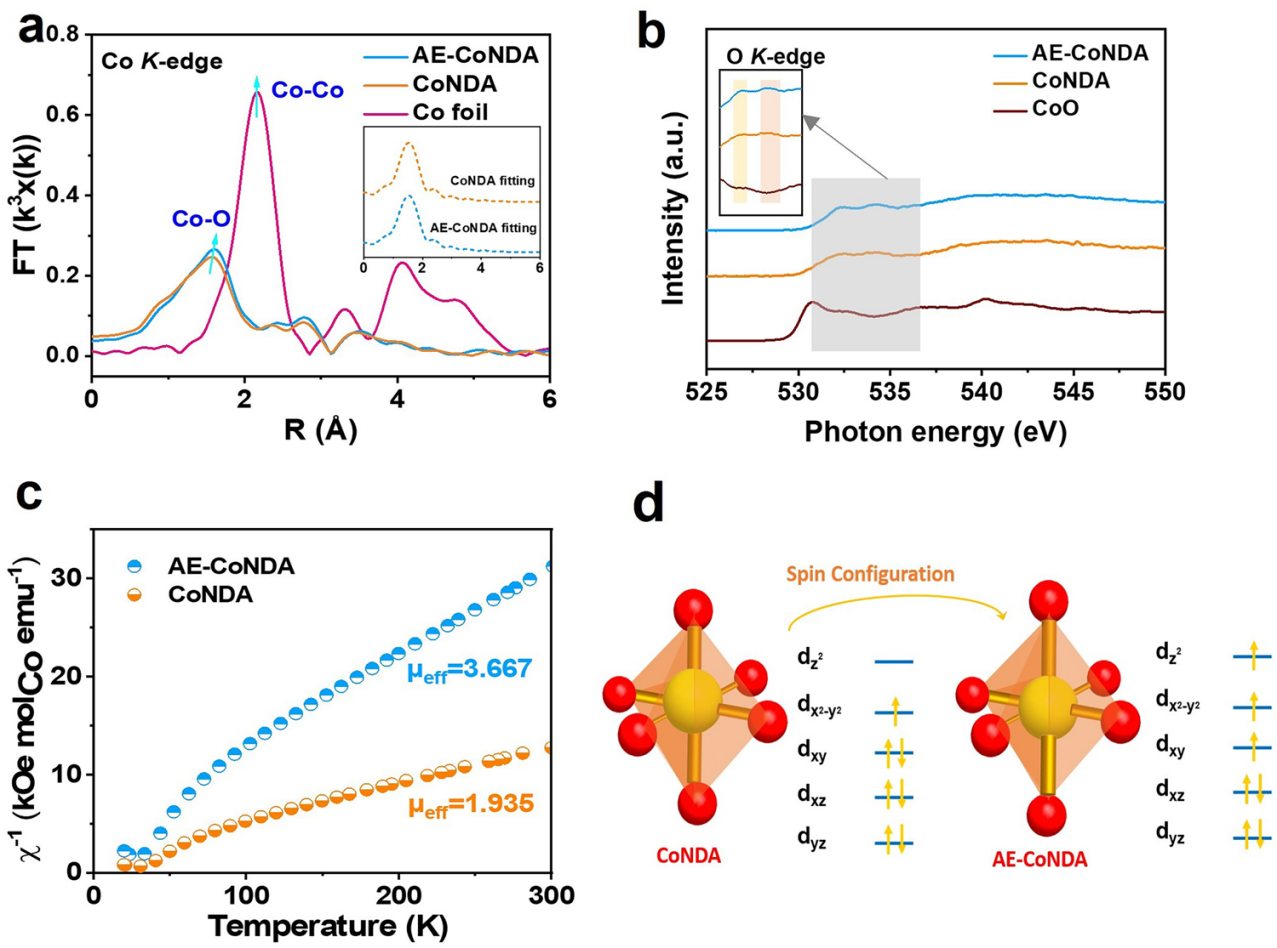
Fine structure analysis and spin state transition of AE-CoNDA. **a** Fourier transformed EXAFS spectra of AE-CoNDA, CoNDA, and Co foil, inset: EXAFS fitting curves in R space of AE-CoNDA and CoNDA. **b** O K-edge XANES spectra of AE-CoNDA, CoNDA, and CoO. **c** Temperature-dependent inverse susceptibilities of AE-CoNDA and CoNDA by the susceptibilities derived from the magnetizations (χ = M/H) follows by Curie–Weiss law. **d** An illustration of 3d-orbitals of AE-CoNDA and CoNDA

Figure 4a shows the corresponding Fourier transforms of extended X-ray absorption fine structure (EXAFS) of AE-CoNDA, which reveals the major peak located at 1.6 Å attributable to the nearest shell coordination of the Co–O bond. It is noteworthy that the Co–O peak of AE-CoNDA shifted toward a higher R direction by about 0.05 Å relative to that of CoNDA, indicating that the bond length of Co–O in the AE-CoNDA was stretched [35]. The stretched Co–O bond is ascribed to partial destruction of CoNDA structure and weakened interaction between Co cations and NDA ligand after acid etching. The EXAFS fitting was performed for the coordination shells in the R range of 1.0–3.0 Å. Amplitude reduction factor S_0_^2^ as determined from fitting was 0.85. The EXAFS fitting results (Table S4) validated that the coordination number of Co–O bonds was determined to be smaller than 6. Additionally, as revealed by the EPR spectra (Fig. S26), an apparent signal at *g*-factor value of 1.86 was observed for AE-CoNDA, whose intensity was stronger than which of CoNDA, indicating more uncoupled Co centers as defects in the framework in AE-CoNDA [40]. EXAFS fitting result and EPR results proved distorted octahedral configurations and unsaturated coordinative Co species of CoO_6_ centers in AE-CoNDA.

To gain more insights into the electron configuration and spin state of AE-CoNDA with stretched Co–O bond length, the O K-edge XAS spectra were further analyzed. As shown in Fig. 4b, the O K-edge XAS spectra consisted of two characteristic peaks between 532.0 and 534.0 eV, arising from the hybridization between unoccupied O 2*p* and Co 3*d* orbitals. Clearly, the intensity of O K-edge from AE-CoNDA was enhanced after acid etching, indicating an increased unoccupied density of states and a strengthening orbital hybridization of O 2*p* and Co 3*d* orbitals. Such enhanced intensity in O K-edge spectra, accompanying with the Co L-edge peak for AE-CoNDA (Fig. 3d), suggests an electron reconfiguration occurred in both the O 2*p* and Co 3*d* orbitals.

It is important to notice that the electronic configuration of Co species primarily dominated the spin state of Co species. To deeply explore the spin state of Co species within AE-CoNDA, we further carried out the zero-field cooling (ZFC) temperature-dependent magnetic susceptibility tests with a magnetic property measurement system (MPMS-VSM) [41]. The total effective magnetic moment (μ_eff_) of AE-CoNDA was obtained through χ^−1^-T liner fitting to calculate the *e*_*g*_ occupancy. In Fig. 4c, the calculated μ_eff_ for AE-CoNDA and CoNDA was 3.667 and 1.935, respectively, and the number of d-orbital unpaired electrons can be further calculated by formula [42]. Thus, the *e*_*g*_ occupancy of Co species raised from approximate 0.98 to 1.84 after Co–O bond length stretched. The calculated *e*_*g*_ occupancy indicated more unpaired d electrons existed in the AE-CoNDA (Fig. 4d), which induced a spin state transition from an intermediate spin (e_g_^1^t_2g_^6^) to a high spin (e_g_^2^t_2g_^5^) of AE-CoNDA [43].

### Theoretical Evaluation of the Enhancement Effect on OER Activity for AE-CoNDA

**Fig. 5:**
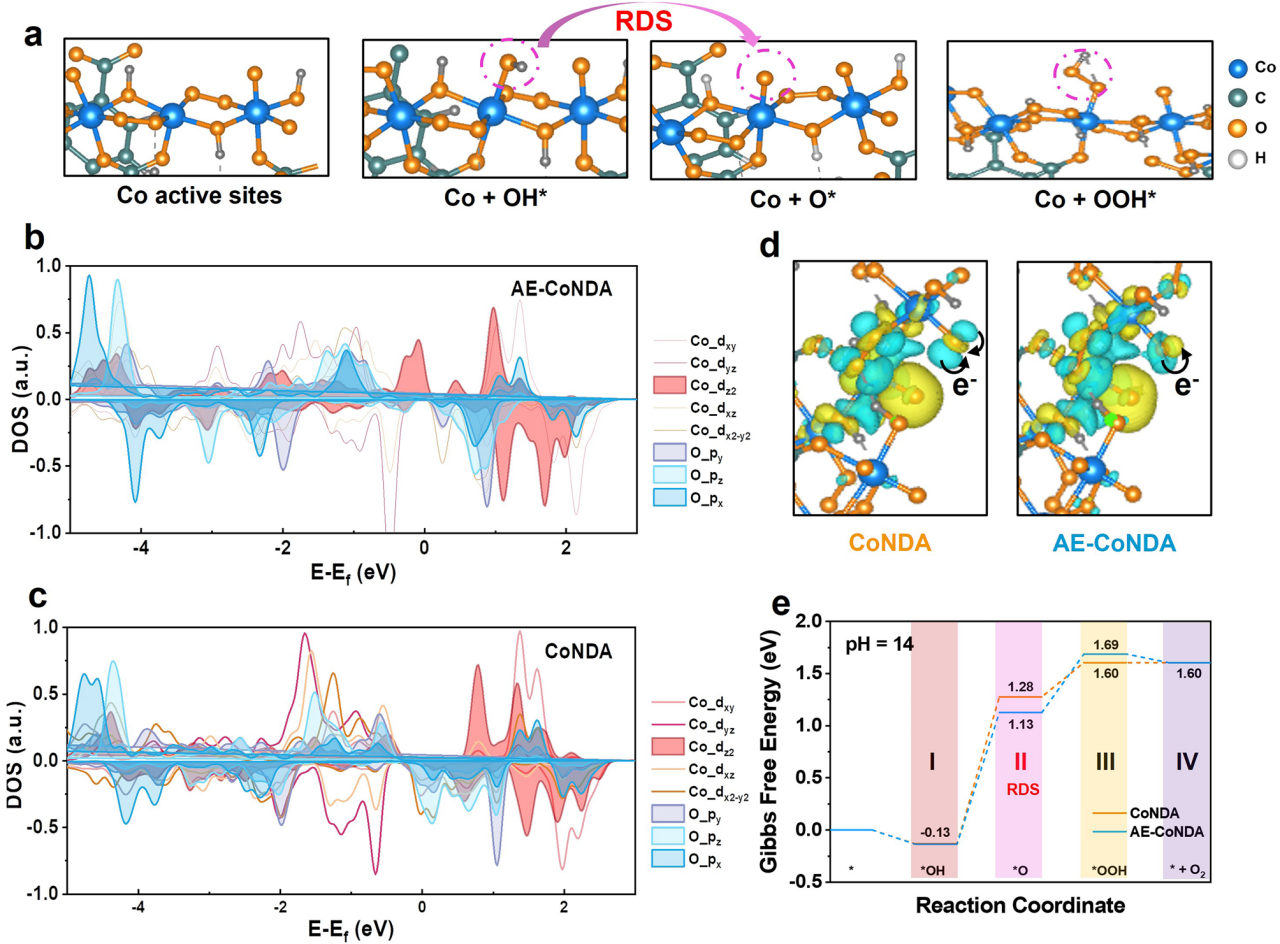
Investigation on correlation between spin states of AE-CoNDA with OER activity.** a** An illustration of AE-CoNDA during OER process including the Co active sites and oxygen-contained intermediates adsorption. Calculated spin-resolved DOS of **b** AE-CoNDA and **c** CoNDA. **d** Calculated charge density difference of AE-CoNDA and CoNDA. The yellow and cyan regions represent electron accumulation and depletion. **e** The free energy diagrams of CoNDA and AE-CoNDA of OER

To give an in-depth understanding of the relationship between the spin state of Co active sites and adsorption ability of oxygen-contained intermediates, we performed DFT calculations. Firstly, a CoNDA model with short bond length and AE-CoNDA model with long bond length were established (Fig. S27). As shown in Fig. 5a, the Co active sites adsorbed oxygen-contained intermediates of OH*, O*, and OOH* through hybridization between the O 2*p* and Co 3*d* orbitals throughout the OER process. Considering that the high spin state of Co active sites was beneficial for Co 3*d* and O 2*p* orbital hybridization to facilitate the adsorption of oxygen-contained intermediates [44], we also calculated the spin-resolved density of spin (DOS). The 3*d*-orbital of Co sites was splitted as *dx*^2^-*y*^2^, *dz*^2^, *dxy*, *dxz*, and *dyz* orbitals in Fig. 5b and c. As for AE-CoNDA (Fig. 5b), the state density of *dz*^2^ (close to the Fermi level) was much stronger than that of CoNDA (Fig. 5c), demonstrating that more electrons transferred between different orbitals in AE-CoNDA. The overlapped area between the Co *dz*^2^ orbital and O 2*p* orbital for AE-CoNDA was larger than that for CoNDA, indicating an enhanced interaction between the Co active sites and oxygen-contained intermediates after the Co–O bond length stretching. These results demonstrated that the bond length stretching facilitated the electron transfer from the *t*_2g_ orbitals to the *e*_g_ orbitals, thus leading to a high spin state [40].

As displayed in Fig. 5d, the AE-CoNDA model showed an accumulated charge of O center in NDA linker from Co active sites with the stretched Co–O bond length compared to control CoNDA, indicating a rapid electron transfer for AE-CoNDA [45]. The specific free energy (ΔG) for each elementary step was calculated to estimate their OER activity at the center Co sites. In Fig. 5e, it can be clearly seen that the elementary steps for the formation of OH* and transition of OOH* to O_2_ desorption were down-hilled, while other reaction steps involved in the OER process for AE-CoNDA and CoNDA were up-hilled. Based on its ΔG diagram, the ΔG value for O* formation at the center Co sites decreased from 1.41 eV for CoNDA to 1.26 eV for AE-CoNDA, revealing that the spin state transition from intermediate spin to high spin led to a decrease in free energy. The application of a bias (U = 0.4 V) also caused a decrease in the ΔG of OH* to O* for AE-CoNDA compared with CoNDA (Fig. S28). Therefore, the formation of the stretched Co–O bond led to a high spin state for the Co active sites, ensuring a readily combination of the O* with the center Co sites. As a result, a reaction path from OH* to O*, which was evidenced as the rate-determined step, was significantly accelerated for AE-CoNDA (Fig. 5e).

## Conclusion

We have developed a highly active AE-CoNDA catalyst with a stretched Co–O bond length and a high spin state. The AE-CoNDA required only a low OER overpotential of 260 mV to reach 10 mA cm^−2^ with a long-term stability. The AE-CoNDA and BiVO_4_ integrated photoanode achieved a photocurrent density of 4.30 mA cm^−2^ at 1.23 V under AM 1.5G irradiation, superior IPCE of 54.0%, ABPE of 1.61%, and prolonged durability in base. Extensive structural characterization verified that the spin state of the center Co sites within the AE-CoNDA was transferred from an intermediate spin state (e_g_^1^t_2g_^6^) to a high spin (e_g_^2^t_2g_^5^) by bond length stretching. The DFT calculations disclosed that the high spin state of AE-CoNDA enhanced the Co 3*d* and O 2*p* orbital hybridization, promoting the reaction kinetics for the RDS (OH* → O*). The in situ electrochemical spectroscopic results demonstrated the bond stretching in AE-CoNDA accelerated the OH* adsorption and facilitated the transfer of photoinduced electrons and holes within the AE-CoNDA@BiVO_4_. This work reports a creative idea for using the bond length adjustment strategy to accelerate the OER kinetics of MOFs-based catalysts, which could be applicable to various photoanodes for a variety of solar-driven applications and renewable energy conversions.

## Supplementary Information

Below is the link to the electronic supplementary material.

Supplementary file1 (DOC 2993 kb)

## References

[1] M. Crespo-Quesada, L.M. Pazos-Outón, J. Warnan, M.F. Kuehnel, R.H. Friend et al., Metal-encapsulated organolead halide perovskite photocathode for solar-driven hydrogen evolution in water. Nat. Commun. **7**, 12555 (2016). 10.1038/ncomms12555 27595974 10.1038/ncomms12555 PMC5025836

[2] X. Yu, V.L. Zholobenko, S. Moldovan, D. Hu, D. Wu et al., Stoichiometric methane conversion to ethane using photochemical looping at ambient temperature. Nat. Energy **5**, 511–519 (2020). 10.1038/s41560-020-0616-7

[3] J.Z. Zhang, E. Reisner, Advancing photosystem II photoelectrochemistry for semi-artificial photosynthesis. Nat. Rev. Chem. **4**, 6–21 (2020). 10.1038/s41570-019-0149-4

[4] T. Bouwens, T.M.A. Bakker, K. Zhu, J. Hasenack, M. Dieperink et al., Using supramolecular machinery to engineer directional charge propagation in photoelectrochemical devices. Nat. Chem. **15**, 213–221 (2023). 10.1038/s41557-022-01068-y 36302868 10.1038/s41557-022-01068-y

[5] V. Andrei, G.M. Ucoski, C. Pornrungroj, C. Uswachoke, Q. Wang et al., Floating perovskite-BiVO_4_ devices for scalable solar fuel production. Nature **608**, 518–522 (2022). 10.1038/s41586-022-04978-6 35978127 10.1038/s41586-022-04978-6

[6] X. You, D. Zhang, X.-G. Zhang, X. Li, J.-H. Tian et al., Exploring the cation regulation mechanism for interfacial water involved in the hydrogen evolution reaction by *in situ* Raman spectroscopy. Nano-Micro Lett. **16**, 53 (2023). 10.1007/s40820-023-01285-1 10.1007/s40820-023-01285-1 PMC10728385 38108934

[7] S. Lyu, C. Guo, J. Wang, Z. Li, B. Yang et al., Exceptional catalytic activity of oxygen evolution reaction via two-dimensional graphene multilayer confined metal-organic frameworks. Nat. Commun. **13**, 6171 (2022). 10.1038/s41467-022-33847-z 36257963 10.1038/s41467-022-33847-z PMC9579180

[8] K. Wang, Y. Wang, B. Yang, Z. Li, X. Qin et al., Highly active ruthenium sites stabilized by modulating electron-feeding for sustainable acidic oxygen-evolution electrocatalysis. Energy Environ. Sci. **15**, 2356–2365 (2022). 10.1039/D1EE03610F

[9] D.Y. Chung, P.P. Lopes, P. Farinazzo Bergamo Dias Martins, H. He, T. Kawaguchi et al., Dynamic stability of active sites in hydr(oxy)oxides for the oxygen evolution reaction. Nat. Energy **5**, 222–230 (2020). 10.1038/s41560-020-0576-y

[10] Z.-F. Huang, J. Song, Y. Du, S. Xi, S. Dou et al., Chemical and structural origin of lattice oxygen oxidation in Co–Zn oxyhydroxide oxygen evolution electrocatalysts. Nat. Energy **4**, 329–338 (2019). 10.1038/s41560-019-0355-9

[11] Y. Tong, Y. Guo, P. Chen, H. Liu, M. Zhang et al., Spin-state regulation of perovskite cobaltite to realize enhanced oxygen evolution activity. Chem **3**, 812–821 (2017). 10.1016/j.chempr.2017.09.003

[12] R.R. Rao, I.E.L. Stephens, J.R. Durrant, Understanding what controls the rate of electrochemical oxygen evolution. Joule **5**, 16–18 (2021). 10.1016/j.joule.2020.12.017

[13] H.-F. Wang, L. Chen, H. Pang, S. Kaskel, Q. Xu, MOF-derived electrocatalysts for oxygen reduction, oxygen evolution and hydrogen evolution reactions. Chem. Soc. Rev. **49**, 1414–1448 (2020). 10.1039/c9cs00906j 32039429 10.1039/c9cs00906j

[14] X. Zhou, B. Fu, L. Li, Z. Tian, X. Xu et al., Hydrogen-substituted graphdiyne encapsulated cuprous oxide photocathode for efficient and stable photoelectrochemical water reduction. Nat. Commun. **13**, 5770 (2022). 10.1038/s41467-022-33445-z 36182949 10.1038/s41467-022-33445-z PMC9526745

[15] J. Xie, F. Wang, Y. Zhou, Y. Dong, Y. Chai et al., Internal polarization field induced hydroxyl spillover effect for industrial water splitting electrolyzers. Nano-Micro. Lett. **16**, 39 (2023). 10.1007/s40820-023-01253-9 10.1007/s40820-023-01253-9 PMC10689691 38032501

[16] X. Ling, F. Du, Y. Zhang, Y. Shen, W. Gao et al., Bimetallic oxyhydroxide *in situ* derived from an Fe_2_Co-MOF for efficient electrocatalytic oxygen evolution. J. Mater. Chem. A **9**, 13271–13278 (2021). 10.1039/D1TA02159A

[17] V. Andrei, R.A. Jagt, M. Rahaman, L. Lari, V.K. Lazarov et al., Long-term solar water and CO_2_ splitting with photoelectrochemical BiOI–BiVO_4_ tandems. Nat. Mater. **21**, 864–868 (2022). 10.1038/s41563-022-01262-w 35618828 10.1038/s41563-022-01262-w

[18] H. Wu, L. Zhang, A. Du, R. Irani, R. van de Krol et al., Low-bias photoelectrochemical water splitting via mediating trap states and small polaron hopping. Nat. Commun. **13**, 6231 (2022). 10.1038/s41467-022-33905-6 36266344 10.1038/s41467-022-33905-6 PMC9585101

[19] L.-W. Wu, C. Liu, Y. Han, Y. Yu, Z. Liu et al., *In situ* spectroscopic identification of the electron-transfer intermediates of photoelectrochemical proton-coupled electron transfer of water oxidation on Au. J. Am. Chem. Soc. **145**, 2035–2039 (2023). 10.1021/jacs.2c11882 36649589 10.1021/jacs.2c11882

[20] X. Zhang, P. Zhai, Y. Zhang, Y. Wu, C. Wang et al., Engineering single-atomic Ni-N_4_-O sites on semiconductor photoanodes for high-performance photoelectrochemical water splitting. J. Am. Chem. Soc. **143**, 20657–20669 (2021). 10.1021/jacs.1c07391 34783534 10.1021/jacs.1c07391

[21] Y. Qi, J. Zhang, Y. Kong, Y. Zhao, S. Chen et al., Unraveling of cocatalysts photodeposited selectively on facets of BiVO_4_ to boost solar water splitting. Nat. Commun. **13**, 484 (2022). 10.1038/s41467-022-28146-6 35079003 10.1038/s41467-022-28146-6 PMC8789891

[22] D. Lee, W. Wang, C. Zhou, X. Tong, M. Liu et al., The impact of surface composition on the interfacial energetics and photoelectrochemical properties of BiVO_4_. Nat. Energy **6**, 287–294 (2021). 10.1038/s41560-021-00777-x

[23] T.W. Kim, K.S. Choi, Nanoporous BiVO_4_ photoanodes with dual-layer oxygen evolution catalysts for solar water splitting. Science **343**, 990–994 (2014). 10.1126/science.1246913 24526312 10.1126/science.1246913

[24] Y. Gao, J. Wang, Y. Yang, J. Wang, C. Zhang et al., Engineering spin states of isolated copper species in a metal–organic framework improves urea electrosynthesis. Nano-Micro Lett. **15**, 158 (2023). 10.1007/s40820-023-01127-0 10.1007/s40820-023-01127-0 PMC10284786 37341868

[25] S. Zhao, C. Tan, C.-T. He, P. An, F. Xie et al., Structural transformation of highly active metal–organic framework electrocatalysts during the oxygen evolution reaction. Nat. Energy **5**, 881–890 (2020). 10.1038/s41560-020-00709-1

[26] J. Yang, Y. Shen, Y. Sun, J. Xian, Y. Long et al., Ir nanoparticles anchored on metal-organic frameworks for efficient overall water splitting under pH-universal conditions. Angew. Chem. Int. Ed. **62**, e202302220 (2023). 10.1002/anie.202302220 10.1002/anie.202302220 36859751

[27] H. Hu, Z. Wang, L. Cao, L. Zeng, C. Zhang et al., Metal-organic frameworks embedded in a liposome facilitate overall photocatalytic water splitting. Nat. Chem. **13**, 358–366 (2021). 10.1038/s41557-020-00635-5 33589788 10.1038/s41557-020-00635-5

[28] J. Xian, S. Li, H. Su, P. Liao, S. Wang et al., Electrosynthesis of α-amino acids from NO and other NO_x_ species over CoFe alloy-decorated self-standing carbon fiber membranes. Angew. Chem. Int. Ed. **62**, e202306726 (2023). 10.1002/anie.202306726 10.1002/anie.202306726 37254227

[29] Z. Jiang, X. Xu, Y. Ma, H.S. Cho, D. Ding et al., Filling metal-organic framework mesopores with TiO_2_ for CO_2_ photoreduction. Nature **586**, 549–554 (2020). 10.1038/s41586-020-2738-2 32906144 10.1038/s41586-020-2738-2

[30] Z. Xue, K. Liu, Q. Liu, Y. Li, M. Li et al., Missing-linker metal-organic frameworks for oxygen evolution reaction. Nat. Commun. **10**, 5048 (2019). 10.1038/s41467-019-13051-2 31695122 10.1038/s41467-019-13051-2 PMC6834668

[31] W. Cheng, X. Zhao, H. Su, F. Tang, W. Che et al., Lattice-strained metal–organic-framework arrays for bifunctional oxygen electrocatalysis. Nat. Energy **4**, 115–122 (2019). 10.1038/s41560-018-0308-8

[32] Y. Sun, Z. Xue, Q. Liu, Y. Jia, Y. Li et al., Modulating electronic structure of metal-organic frameworks by introducing atomically dispersed Ru for efficient hydrogen evolution. Nat. Commun. **12**, 1369 (2021). 10.1038/s41467-021-21595-5 33649349 10.1038/s41467-021-21595-5 PMC7921655

[33] K. Liu, J. Fu, Y. Lin, T. Luo, G. Ni et al., Insights into the activity of single-atom Fe-N-C catalysts for oxygen reduction reaction. Nat. Commun. **13**, 2075 (2022). 10.1038/s41467-022-29797-1 35440574 10.1038/s41467-022-29797-1 PMC9018836

[34] F. Cheng, X. Peng, L. Hu, B. Yang, Z. Li et al., Accelerated water activation and stabilized metal-organic framework via constructing triangular active-regions for ampere-level current density hydrogen production. Nat. Commun. **13**, 6486 (2022). 10.1038/s41467-022-34278-6 36309525 10.1038/s41467-022-34278-6 PMC9617936

[35] F. He, Q. Zheng, X. Yang, L. Wang, Z. Zhao et al., Spin-state modulation on metal-organic frameworks for electrocatalytic oxygen evolution. Adv. Mater. **35**, e2304022 (2023). 10.1002/adma.202304022 37358536 10.1002/adma.202304022

[36] L. Zhang, R. Long, Y. Zhang, D. Duan, Y. Xiong et al., Direct observation of dynamic bond evolution in single-atom Pt/C_3_ N_4_ catalysts. Angew. Chem. Int. Ed. Engl. **59**, 6224–6229 (2020). 10.1002/anie.201915774 31922641 10.1002/anie.201915774

[37] W.H. Lee, M.H. Han, Y.J. Ko, B.K. Min, K.H. Chae et al., Electrode reconstruction strategy for oxygen evolution reaction: maintaining Fe-CoOOH phase with intermediate-spin state during electrolysis. Nat. Commun. **13**, 605 (2022). 10.1038/s41467-022-28260-5 35105874 10.1038/s41467-022-28260-5 PMC8807628

[38] H. Tao, Y. Xu, X. Huang, J. Chen, L. Pei et al., A general method to probe oxygen evolution intermediates at operating conditions. Joule **3**, 1498–1509 (2019). 10.1016/j.joule.2019.03.012

[39] S. Chibani, C. Michel, F. Delbecq, C. Pinel, M. Besson, On the key role of hydroxyl groups in platinum-catalysed alcohol oxidation in aqueous medium. Catal. Sci. Technol. **3**, 339–350 (2013). 10.1039/C2CY20363D

[40] X. Kang, K. Lyu, L. Li, J. Li, L. Kimberley et al., Integration of mesopores and crystal defects in metal-organic frameworks via templated electrosynthesis. Nat. Commun. **10**, 4466 (2019). 10.1038/s41467-019-12268-5 31578368 10.1038/s41467-019-12268-5 PMC6775123

[41] F. He, Y. Zhao, X. Yang, S. Zheng, B. Yang et al., Metal-organic frameworks with assembled bifunctional microreactor for charge modulation and strain generation toward enhanced oxygen electrocatalysis. ACS Nano **16**, 9523–9534 (2022). 10.1021/acsnano.2c02685 35616603 10.1021/acsnano.2c02685

[42] J.-Y. Zhang, Y. Yan, B. Mei, R. Qi, T. He et al., Local spin-state tuning of cobalt–iron selenide nanoframes for the boosted oxygen evolution. Energy Environ. Sci. **14**, 365–373 (2021). 10.1039/D0EE03500A

[43] B.E. Van Kuiken, M. Khalil, Simulating picosecond iron K-edge X-ray absorption spectra by *ab initio* methods to study photoinduced changes in the electronic structure of Fe(II) spin crossover complexes. J. Phys. Chem. A **115**, 10749–10761 (2011). 10.1021/jp2056333 21846088 10.1021/jp2056333

[44] G. Zhou, P. Wang, H. Li, B. Hu, Y. Sun et al., Spin-sate reconfiguration induced by alternating magnetic field for efficient oxygen evolution reaction. Nat. Commun. **12**, 4827 (2021). 10.1038/s41467-021-25095-4 34376676 10.1038/s41467-021-25095-4 PMC8355122

[45] J. Suntivich, K.J. May, H.A. Gasteiger, J.B. Goodenough, Y. Shao-Horn, A perovskite oxide optimized for oxygen evolution catalysis from molecular orbital principles. Science **334**, 1383–1385 (2011). 10.1126/science.1212858 22033519 10.1126/science.1212858

