# Supplementary material for: Accelerating Oxygen Electrocatalysis Kinetics on Metal–Organic Frameworks via Bond Length Optimization
Source: Nanomicro Lett. 2024 Apr 19;16:175. doi: 10.1007/s40820-024-01382-9 (PMC11031554; doi:10.1007/s40820-024-01382-9)
Supplement: Supplementary file 1 — Supplementary file1 (DOC 2993 kb) [file 40820_2024_1382_MOESM1_ESM.doc]

Supporting Information for

**Accelerating Oxygen Electrocatalysis Kinetics on Metal-Organic Frameworks via Bond Length Optimization**

Fan He^1^, Yingnan Liu^1^, Xiaoxuan Yang^1^, Yaqi Chen^1^, Cheng-Chieh Yang^5^, Chung-Li Dong^5^, Qinggang He^1^, Bin Yang^1^, Zhongjian Li^1^, Yongbo Kuang^3^, Lecheng Lei^1^, Liming Dai^6^, Yang Hou^1,2,4^,*

^1^ Key Laboratory of Biomass Chemical Engineering of Ministry of Education, College of Chemical and Biological Engineering, Zhejiang University, Hangzhou 310027, P. R. China

^2^ Institute of Zhejiang University - Quzhou, Quzhou 324000, P. R. China

^3^ Ningbo Institute of Materials Technology and Engineering, Chinese Academy of Sciences, Ningbo 315201, P. R. China

^4^ School of Biological and Chemical Engineering, NingboTech University, Ningbo, 315100, P. R. China

^5^ Department of Physics, Tamkang University, New Taipei, 25137 Taiwan, P. R. China

^6^ Australian Carbon Materials Centre (A-CMC), School of Chemical Engineering, University of New South Wales, Sydney, New South Wales 2051, Australia

*Corresponding author. E-mail: [yhou@zju.edu.cn](mailto:yhou@zju.edu.cn) (Yang Hou)

**S1 Experimental Section**

**S1.1 Synthesis of CoNDA**

2,6-NDA (172.9 mg) and CoCl_2_·6H_2_O (190.2 mg) were dissolved in a mixed solution containing 32 mL dimethyl formamide (DMF), 2 mL ethanol, and 2 mL deionized water. Afterwards, 1.0 mL triethylamine (TEA) was injected into above mixed solution with vigorous stirring to form a uniform colloidal suspension. The formed suspension was sealed and further ultrasonicated for 300 min under a power of 70 kHz at room temperature. Subsequently, the precipitates were washed with DMF and ethanol for three times, and dried in vacuum oven at 60 ^o^C.

**S1.2 Synthesis of AE-CoNDA with different concentrations of acetic acid etching**

2,6-NDA (172.9 mg) and CoCl_2_·6H_2_O (190.2 mg) were dissolved in a mixed solution containing 32 mL dimethyl formamide (DMF), 2 mL ethanol, and 2 mL deionized water. Afterwards, 1.0 mL triethylamine (TEA) was injected into above mixed solution with vigorous stirring to form a uniform colloidal suspension. The formed suspension was sealed and further ultrasonicated for 300 min under a power of 70 kHz at room temperature. The obtained sample was further immersed in 0.1 mM, 0.2 mM, 0.5 mM or 1.0 mM acetic acid solution for etching. The obtained precipitate was washed with DMF and ethanol for three times, and dried in vacuum oven at 60 ^o^C.

**S1.3 Synthesis of AE-CoNDA with different etching time by 0.2 mM acetic acid**

2,6-NDA (172.9 mg) and CoCl_2_·6H_2_O (190.2 mg) were dissolved in a mixed solution containing 32 mL dimethyl formamide (DMF), 2 mL ethanol, and 2 mL deionized water. Afterwards, 1.0 mL triethylamine (TEA) was injected into above mixed solution with vigorous stirring to form a uniform colloidal suspension. The formed suspension was sealed and further ultrasonicated for 300 min under a power of 70 kHz at room temperature. The obtained sample was further immersed in 0.2 mM acetic acid solution for 0.5 h, 1 h, 2 h or 3 h etching. The obtained precipitate was washed with DMF and ethanol for three times, and dried in vacuum oven at 60 ^o^C.

**S1.4 Synthesis of BiVO_4_**

The BiOI precursor was electrodeposited on an FTO substrate in a three-electrode cell, which was prepared by a 100 mL of HNO_3_ mixture solution containing 0.0075 M Bi(NO_3_)_3_, 0.4 M NaI, and 45 mL of 0.3 M p-benzoquinone in ethanol. To convert the BiOI precursor into BiVO_4_, the as-prepared BiOI electrode was immersed in a 0.4 M C_15_H_21_O_6_V dissolved in dimethylsulfoxide (DMSO) for 20 s, and then transferred into a muffle furnace preheated to 120 °C, then hold at 450 °C for 1 h. The resulting BiVO_4_ electrode was soaked in a 0.05 M NaOH solution for 5 min then rinsed with DI water and dried in air for further use^7^.

**S1.5 Synthesis of CoNDA@BiVO_4_**

10 mg of CoNDA catalyst was dispersed in 5 mL ethanol, then sprayed on the as-prepared BiVO_4_. Then, the above electrode was annealed at 100 °C for 30 min to obtain the CoNDA@BiVO_4_ photoanode.

**S1.6 Synthesis of AE-CoNDA@BiVO_4_**

10 mg of AE-CoNDA catalyst was dispersed in 5 mL ethanol, and sprayed on the as-prepared BiVO_4_. Then, the above electrode was annealed at 100 °C for 30 min to obtain the AE-CoNDA@BiVO_4_ photoanode.

**S1.7 PEC-OER measurements**

The charge transfer efficiencies were estimated as functions of applied potential by using Na_2_SO_3_ as hole scavenger under AM 1.5 G irradiation. We assume that the oxidation kinetics of Na_2_SO_3_ is very fast and its charge transfer efficiency is 100%. Therefore, the ratio of photocurrent densities measured in H_2_O and Na_2_SO_3_ can be used to calculate charge transfer efficiency. The ABPE that is the conversion efficiency from light energy to chemical energy, is calculated as:

ABPE= [(1.23-V_app_) × (J_light_-J_dark_)/P_light_] × 100%

where V_app_ is the applied potential vs. RHE, J_dark_ and J_light_ are the respective current densities in dark and under AM 1.5 G irradiation, and P_light_ is the power density of AM 1.5 G (100 mW cm^-2^).

The EIS was performed at a DC bias of 0.9 V in the frequency range of 10^5^-0.01 Hz with an AC voltage of 10 mV.

IPCE was calculated using the equation, IPCE = ((1240/λ) × (J_light_ - J_dark_)/P_λ_)) × 100%

The electrolysis was carried out with a constant potential at 0.7 V vs. RHE, and the actual amount of O_2_ generated was determined by gas chromatography (GC-2014 SHIMADZU) every ca. 30 min. The theoretical amount of produced O_2_ can be obtained by converting the charge passed to generated gas (μmol) according to Faraday’s Law. The Faradaic efficiency was calculated according to the equation: FE (%) = O_2_ (actual) / O_2_ (theoretical) × 100%.

**S1.8 Computational methods**

The (1 × 2) surface of bulk CoNDA (100) was chosen, which contains 16 Co atoms, 83 O atoms, 96 C atoms and 64 H atoms. A vacuum slab of about 15 Å was maintained in the super-cell configuration that was large enough for the calculations. To simulate the tensile state of structure, tensile CoNDA structure was constructed by expanding the lattice parameters of the pristine CoNDA in three directions by 5%.

The reaction pathways for OER under alkaline environment are simulated as follows:

*OH→OH* S1

OH* + (OH^-^ + e^-^) →O* + H_2_O S2

O* + (OH^-^ + e^-^) →OOH* S3

OOH* + (OH^-^ + e^-^) → O_2_↑+H_2_O S4

In these equations, the * denotes the catalytic active site on surface; the OH*, O* and OOH* denote the reaction intermediates with adsorbed groups of OH, O, and OOH, respectively.

The equation for the Gibbs free energy calculations is as follows:

G = E_o_ + E_zpv_ - TS + G_U_ S5

where E_0_ is the total energy obtained from DFT calculation, E_zpv_, T and S stand for the zero point vibration energy, temperature (298.15 K) and entropy corrections, respectively. G_U_ is depended on the electrode potential V and electron charge e.

**S2 Supplementary Figures**

**
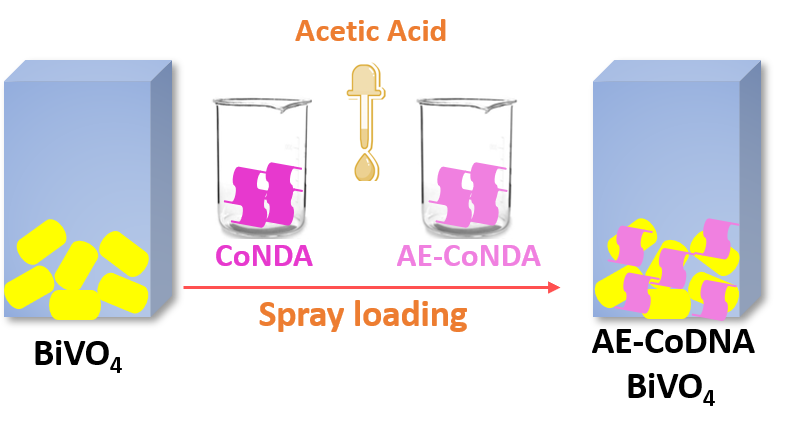
**

**Fig. S1** Schematic illustration of the synthesis process of AE-CoNDA@BiVO_4_

**
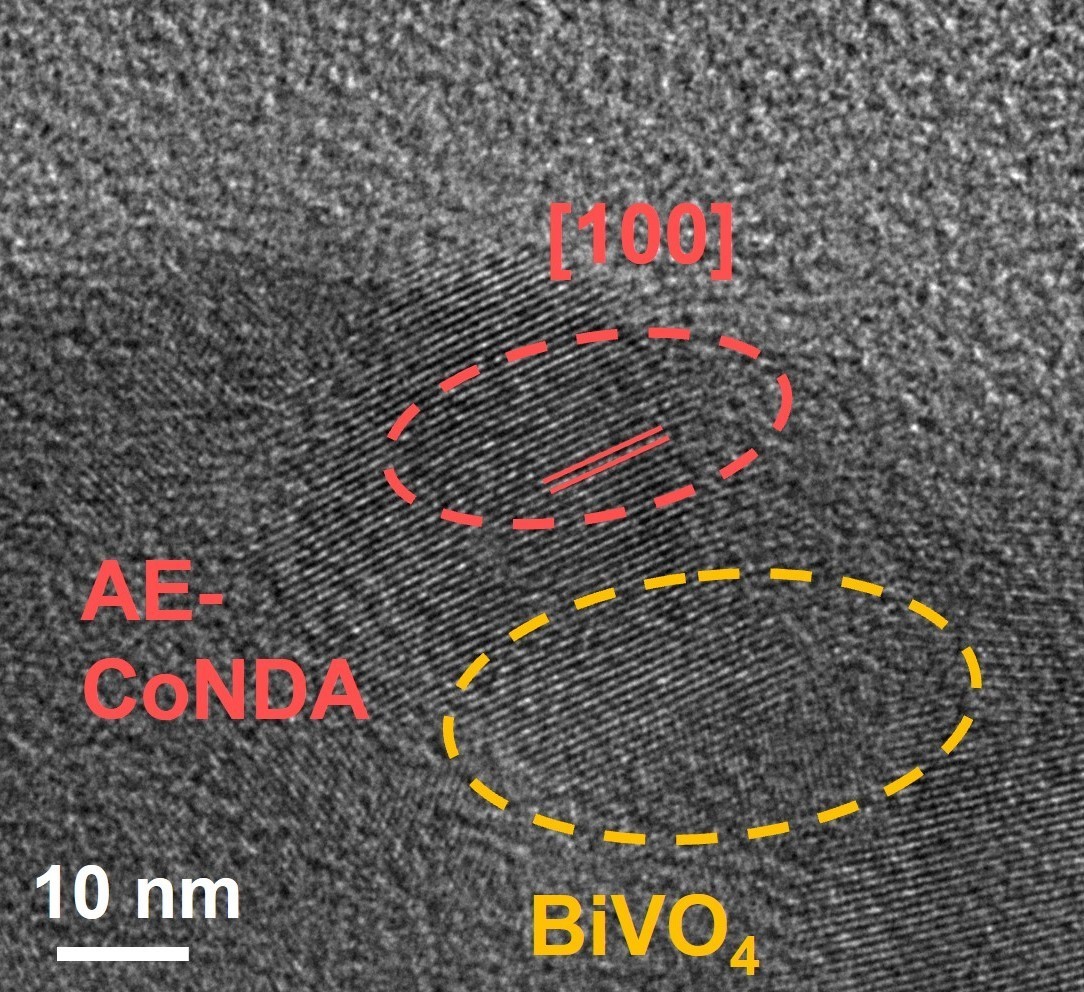
**

**Fig. S2** HR-TEM image of AE-CoNDA@BiVO_4_

**
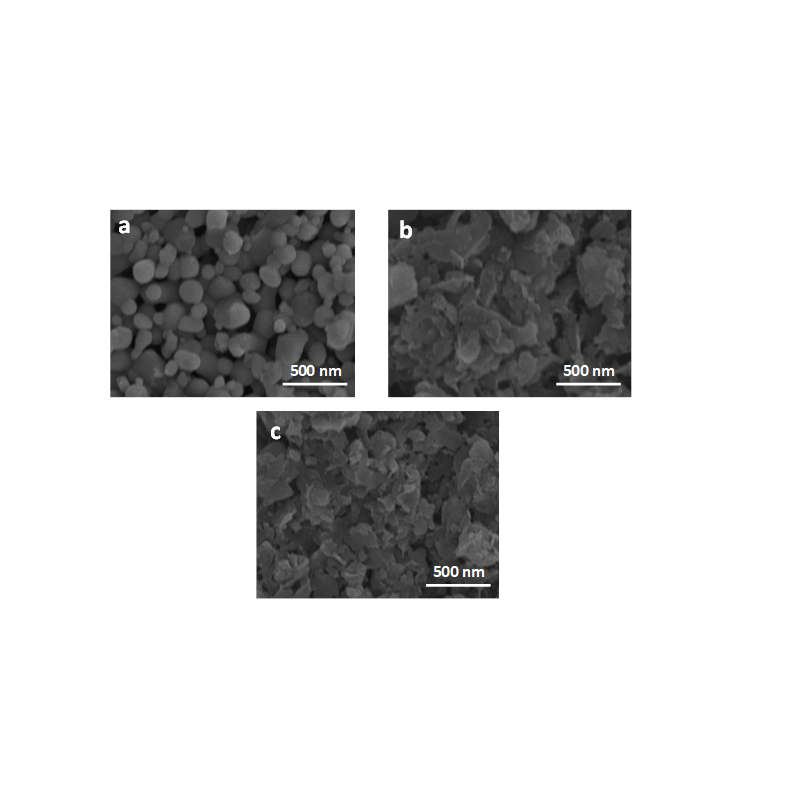
**

**Fig. S3** FESEM images of BiVO_4_ (**a**), CoNDA@BiVO_4_ (**b**), and AE-CoNDA@BiVO_4_ (**c**)


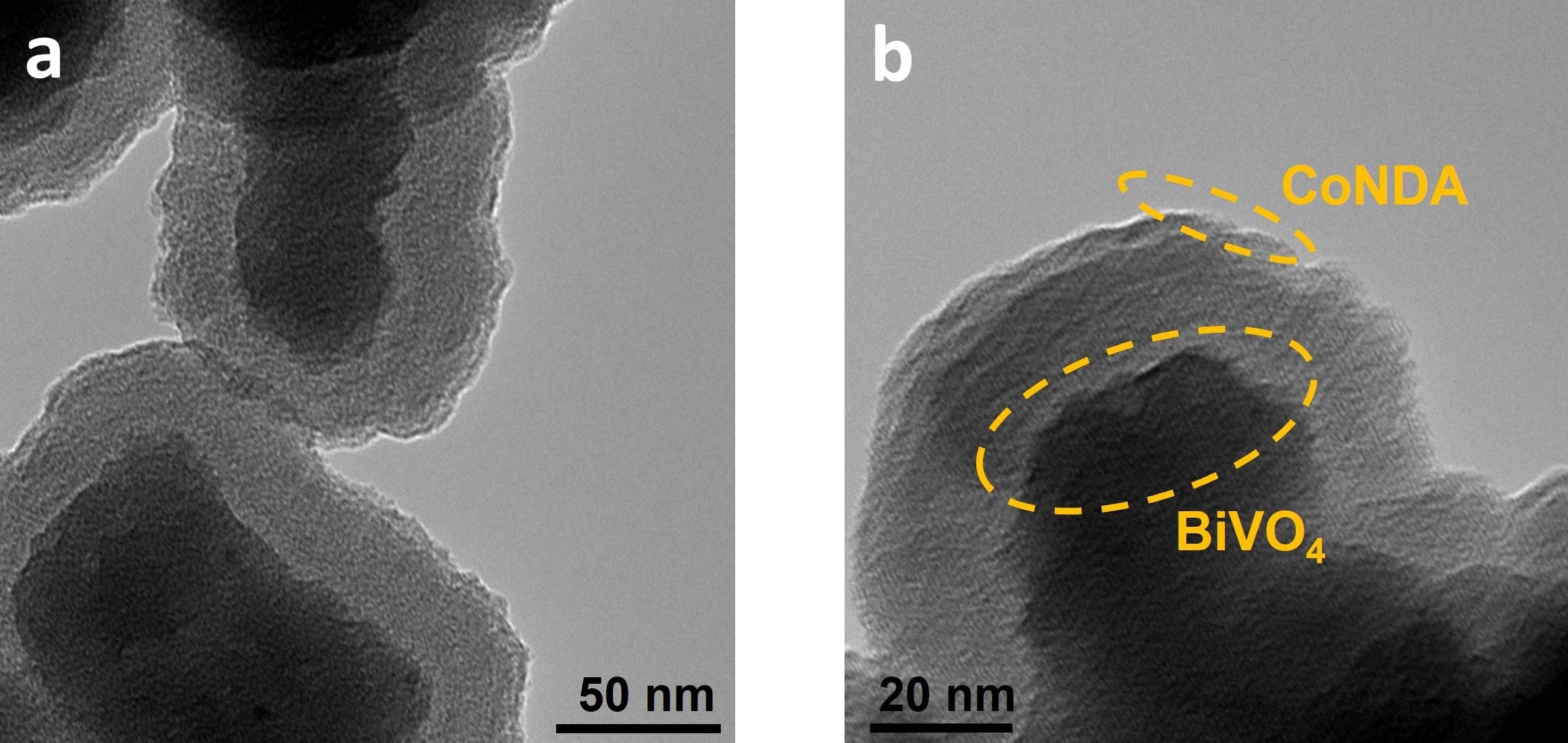


**Fig. S4** TEM images of BiVO_4_ **(a)** and CoNDA@BiVO_4_ **(b)**


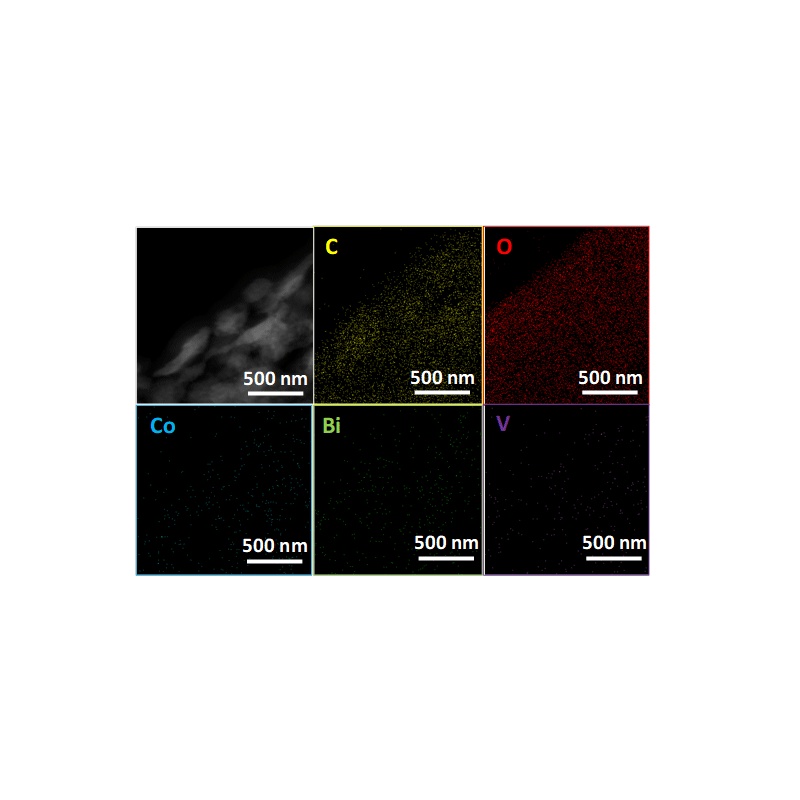


**Fig. S5** HR-TEM and EDX elemental mapping images of AE-CoNDA@BiVO_4_

**Fig. S6** XRD patterns of AE-CoNDA@BiVO_4_, CoNDA@BiVO_4_ and BiVO_4_

**Fig. S7** Raman spectra of BiVO_4_, CoNDA@BiVO_4_, and AE-CoNDA@BiVO_4_

**Fig. S8** Polarization curves of BiVO_4_ (**a**) and AE-CoNDA@BiVO_4_ with different spray layers of AE-CoNDA (**b-f**), without adding Na_2_SO_3_ under chopped AM 1.5G irradiation

**Fig. S9** EIS Nyquist plots of AE-CoNDA@BiVO_4_, CoNDA@BiVO_4_, and BiVO_4_ under AM 1.5G light illumination

**Fig. S10** Polarization curves of BiVO_4_, CoNDA@BiVO_4_, and AE-CoNDA@BiVO_4_ with or without Na_2_SO_3_ under AM 1.5G irradiation

To understand the positive effect of AE-CoNDA on the promotion of photogenerated charge carrier separation, the charge transfer efficiencies (η_transfer_= J_H2O_/J_Na2SO3_) and the charge transport efficiencies (η_transport_ = J_Na2SO3_/J_abs_) of the AE-CoNDA are decoupled and quantified by using Na_2_SO_3_ as hole scavenger. The specific efficiency is calculated by polarization curves [S1].

**Fig. S11** Solar irradiance of AM 1.5G and UV-Vis DRS of BiVO_4_, CoNDA@BiVO_4_, and AE-CoNDA@BiVO_4_. The overlapped area is Jabs

**Fig. S12** Charge transfer efficiencies of BiVO_4_, CoNDA@BiVO_4_, and AE-CoNDA@BiVO_4_

At 1.23 V, AE-CoNDA@BiVO_4_ delivers a much higher η_transfer_ of 90.0% than CoNDA@BiVO_4_ (81.1%) and bare BiVO_4_ (62.5%), illustrating that AE-CoNDA effectively weaken surface charge recombination and improve charge transfer from BiVO_4_ to electrolyte, thus facilitating PEC-OER kinetics.

**Fig. S13** Charge transport efficiencies of BiVO_4_, CoNDA@BiVO_4_, and AE-CoNDA@BiVO_4_

**Fig. S14** Stability of CoNDA@BiVO_4_ at 1.5 mA cm^-2^ and BiVO_4_ at 1.0 mA cm^-2^ under AM 1.5G irradiation

**Fig. S15** *Operando* irradiated high-resolution Co 2*p* (**a**) and V 2*p* (**b**) XPS spectra of AE-CoNDA@BiVO_4_

After light illumination, the Co 2*p* XPS peaks of AE-CoNDA@BiVO_4_ evidently shifted to higher binding energy compared with AE-CoNDA, while V 2*p* XPS peak shifted to lower binding energy, which strongly claim the photo-induced holes transferred from BiVO_4_ to AE-CoNDA cocatalyst, achieving a favorable charge transfer of bare BiVO_4_ photoanode.

**Fig. S16** (**a**) Polarization curves of CoNDA with different concentrations of acid after acid etching. (**b**) Polarization curves of CoNDA with different acid etching time

With the concentration of acetic acid increases, the OER performance of AE-CoNDA is significantly improved, which proves that the proper concentration for acid etching could efficiently increase the OER activity. After the concentration of acetic acid exceeds 0.2 mM, the OER performance of AE-CoNDA is reduced, which means that the high concentration of acetic acid induces a poor OER performance, which is attributed to the damage of the original structure of CoNDA. Thus, the optimal concentration of acetic acid for acid etching is determined to be 0.2 mM.

To explore the optimal acid etching time, a series of AE-CoNDA samples with different acid etching times were prepared. With an increase in the acid etching time, the OER performance of AE-CoNDA is improved, which proves that the proper acid etching treatment is favorable for electrocatalytic OER. When the acid etching time is longer than 1 h, the OER performance of AE-CoNDA is reduced, indicating that the long acid etching time will destroy the pristine CoNDA structure, and the short acid etching time is insufficient to regulate the Co-O bond in the pristine CoNDA structure. Therefore, the optimal acid etching time is determined to be 1 h.

**Fig. S17** XRD patterns of CoNDA with different concentrations of acetic acid after acid etching

**Fig. S18** XRD patterns of CoNDA with different acetic acid etching time

**Fig. S19** EIS Nyquist plots and equivalent electrical circuit fitting of CoNDA and AE-CoNDA

**Fig. S20** Calculation for internal and external voltametric charge densities. Linear plot of (**a**) q^-1^ vs. v^0.5^ and (**c**) q vs. v^-0.5^ of CoNDA, and AE-CoNDA (**b**) and (**d**) are enlarged graphs of the red dotted boxes in (**a**) and (**c**), respectively

**Fig. S21** CV curves of (**a**) CoNDA and (**b**) AE-CoNDA at different scan rates of 10 mV s^-1^ to 50 mV s^-1^ in 1.0 M KOH

**Fig. S22** C*dl* of CoNDA and AE-CoNDA (**a**). Polarization curves of CoNDA and AE-CoNDA normalized by ECSA (**b**)

**Fig. S23** Chronopotentiometric durability of CoNDA at 1.5 V

**Fig. S24** (**a**) XRD patterns and (**b**) Raman spectra of AE-CoNDA before and after OER process

**Fig. S25** High-resolution C 1*s* XPS spectra of CoNDA and AE-CoNDA

**Fig. S26** EPR spectra of CoNDA and AE-CoNDA

**Fig. S27** Crystal structures of AE-CoNDA model after bond length regulation (**a**) and CoNDA models (**b**)

**Fig. S28** The free energy diagrams of CoNDA and AE-CoNDA of OER at U = 0.4 V

**Table S1** Comparison of PEC-OER performances of AE-CoNDA@BiVO_4_ with other reported Co-based cocatalyst loaded on BiVO_4_

| Photoanode | Photocurrent density (mA cm^-2^) | | IPCE (%) | ABPE  (%) | Stability  (h) | Refs. |
| --- | --- | --- | --- | --- | --- | --- |
|  | 0.6 V | 1.23 V |  |  |  |  |
| **AE-CoNDA@BiVO_4_** | **2.4** | **4.3** | **52.5** | **1.6** | **20** | **This work** |
| CoPiB/BiVO_4_/ZnO | 0.1 | 3.5 | 40 | 1.1 | 3 | [S2] |
| NiCo-LDH/BiVO_4_ | 0.6 | 3.4 | 59 | 0.66 | 3 | [S3] |
| NiO/CoOx/BiVO_4_ | 2.5 | 3.5 | 60 | 1.5 | 16 | [S4] |
| Mo-BiVO_4_ | 3.4 | 4.98 | 71 | 0.65 | 10 | [S5] |
| CoNi-MOF/BiVO_4_ | 0.3 | 3.2 | 32 | 1.8 | 3 | [S6] |
| CoBDA FcCA/BiVO_4_ | 2.8 | 3.5 | 44.3 | 0.67 | 2.7 | [S7] |

**Table S2** EIS fitting data of CoNDA and AE-CoNDA cocatalyst in 1.0 M KOH in this work

| Sample | Potential (V) | R_ct_ (kΩ) |  | Sample | Potential (V) | R_ct_ (kΩ) |
| --- | --- | --- | --- | --- | --- | --- |
|  | 1.20 | 7.653 |  |  | 1.20 | 2.355 |
|  | 1.25 | 6.127 |  |  | 1.25 | 1.773 |
|  | 1.30 | 3.302 |  |  | 1.30 | 1.032 |
| CoNDA | 1.35 | 2.177 |  | AE-CoNDA | 1.35 | 0.667 |
|  | 1.40 | 1.489 |  |  | 1.40 | 0.128 |
|  | 1.45 | 0.995 |  |  | 1.45 | 0.099 |
|  | 1.50 | 0.623 |  |  | 1.50 | 0.064 |
|  | 1.55 | 0.356 |  |  | 1.55 | 0.043 |
|  | 1.60 | 0.088 |  |  |  |  |

**Table S3 Comparison of OER performances of AE-CoNDA with other reported non-carbonized Co-MOF-based OER catalysts in alkaline media**

| Catalyst | Overpotential @10 mA cm^-2^ | Tafel slope | Substrate | Refs. |
| --- | --- | --- | --- | --- |
| **AE-CoNDA** | **260 mV** | **62 mV dec^-1^** | **GCE** | **This work** |
| CoP-InNC@CNT | 270 mV | 84 mV dec^-1^ | GCE | [S8] |
| CoFe20@CC | 286 mV | 58.8 mV dec^-1^ | GCE | [S9] |
| A_2.7_B-MOF-FeCo_1.6_ | 288 mV | 39 mV dec^-1^ | GCE | [S10] |
| Co_3_O_4_C-NA | 290 mV | 70 mV dec^-1^ | GCE | [S11] |
| FeCo-MNS-1.0 | 298 mV | 21.6 mV dec^-1^ | GCE | [S12] |
| NCF-MOF | 320 mV | 49 mV dec^-1^ | RDE | [S13] |
| Co_3_O_4_/HNCP-40 | 333 mV | 69 mV dec^-1^ | GCE | [S14] |
| MAF-X27-OH | 387 mV | 60 mV dec^-1^ | GCE | [S15] |

**Table S4 Structural parameters of CoNDA and AE-CoNDA nanosheets extracted from the EXAFS fitting (S_0_^2^ = 0.85)**

| Sample | Scattering pair | CN | R(Å) | σ^2^(10^-3^Å^2^) | ΔE_0_(eV) | R |
| --- | --- | --- | --- | --- | --- | --- |
| CoNDA | Co-O | 5.68(4) | 2.07 | 11(8) | -1.04(5) | 0.002 |
| AE-CoNDA | Co-O | 5.47(5) | 2.09 | 13(8) | -1.41(5) | 0.002 |

S_0_^2^ is the amplitude reduction factor; CN is the coordination number; R is interatomic distance (the bond length between central atoms and surrounding coordination atoms); σ^2^ is Debye-Waller factor (a measure of thermal and static disorder in absorber-scatterer distances); ΔE_0_ is edge-energy shift (the difference between the zero kinetic energy value of the sample and that of the theoretical model). R factor is used to value the goodness of the fitting.

**Table S5 ICP-MS elemental analysis results of Co content for CoNDA, and AE-CoNDA with different concentrations of acetic acid etching for 1 h**

| Samples | Co (wt%) |
| --- | --- |
| CoNDA | 23.07 |
| AE-CoNDA-0.1 mM | 22.88 |
| AE-CoNDA-0.2 mM | 21.98 |
| AE-CoNDA-0.5 mM | 18.41 |
| AE-CoNDA-1.0 mM | 16.22 |

**Table S6 ICP-MS elemental analysis results of Co content for CoNDA, and AE-CoNDA with different acid etching time by 0.2 mM acetic acid**

| Samples | Co (wt%) |
| --- | --- |
| CoNDA | 23.07 |
| AE-CoNDA-0.5 h | 22.43 |
| AE-CoNDA-1 h | 21.98 |
| AE-CoNDA-2 h | 20.15 |
| AE-CoNDA-3 h | 18.27 |

**Supplementary References**

1. Y. Hou, M. Qiu, M. Kim, P. Liu, G. Nam, et al., Atomically dispersed nickel-nitrogen-sulfur species anchored on porous carbon nanosheets for efficient water oxidation. Nat. Commun. **10**, 1392 (2019). https://doi.org/10.1038/s41467-019-09394-5.
2. J. Yang, J. Wu, Low-potential driven fully-depleted BiVO_4_/ZnO heterojunction nanodendrite array photoanodes for photoelectrochemical water splitting. Nano Energy **32**, 232-240 (2017). https://doi.org/10.1016/j.nanoen.2016.12.039.
3. H. She, P. Yue, X. Ma, J. Huang, L. Wang, et al., Fabrication of BiVO_4_ photoanode cocatalyzed with NiCo-layered double hydroxide for enhanced photoactivity of water oxidation. Appl. Catal. B: Environ. **263**, 118280 (2020). https://doi.org/10.1016/j.apcatb.2019.118280.
4. M. Zhong, T. Hisatomi, Y. Kuang, J. Zhao, M. Liu, et al., Surface modification of CoO_x_ loaded BiVO_4_ photoanodes with ultrathin p-type NiO layers for improved solar water oxidation. J. Am. Chem. Soc. **137**, 5053-5060 (2015). https://doi.org/10.1021/jacs.5b00256.
5. K. Ye, H. Li, D. Huang, S. Xiao, W. Qiu, et al., Enhancing photoelectrochemical water splitting by combining work function tuning and heterojunction engineering. Nat. Commun. **10**, 3687 (2019). https://doi.org/10.1038/s41467-019-11586-y.
6. S. Zhou, K. Chen, J. Huang, L.Wang, M. Zhang, et al., Preparation of heterometallic CoNi-MOFs-modified BiVO_4_: a steady photoanode for improved performance in photoelectrochemical water splitting. Appl. Catal. B: Environ. **266**, 118513 (2020). https://doi.org/10.1016/j.apcatb.2019.118513.
7. F. He, Y. Zhao, X. Yang, S. Zheng, B. Yang, et al., Metal-Organic frameworks with assembled bifunctional microreactor for charge modulation and strain generation toward enhanced oxygen electrocatalysis. ACS Nano **6**, 9523-9534 (2022). https://doi.org/10.1021/acsnano.2c02685.
8. L. Chai, Z. Hu, X. Wang, Y. Xu, L. Zhang, et al., Stringing bimetallic metal-organic framework-derived cobalt phosphide composite for high-efficiency overall water splitting. Adv. Sci. **7**, 1903195 (2020). https://doi.org/10.1002/advs.201903195.
9. C. Hou, L. Zou, Q. Xu, A hydrangea-like superstructure of open carbon cages with hierarchical porosity and highly active metal sites. Adv. Mater. **31**, 1904689 (2019). https://doi.org/10.1002/adma.201904689.
10. Z. Xue, Y. Li, Y. Zhang, W. Geng, B. Jia, et al., Modulating electronic structure of metal-organic framework for efficient electrocatalytic oxygen evolution. Adv. Energy Mater. **8**, 1801564 (2018). https://doi.org/10.1002/aenm.201801564.
11. T. Ma, S. Dai, M. Jaroniec, S. Qiao, Metal-organic framework derived hybrid Co_3_O_4_-carbon porous nanowire arrays as reversible oxygen evolution electrodes. J. Am. Chem. Soc. **136**, 13925-13931 (2014). https://doi.org/10.1021/ja5082553.
12. L. Zhuang, L. Ge, H. Liu, Z. Jiang, Y. Jia, et al., A surfactant-free and scalable general strategy for synthesizing ultrathin two-dimensional metal-organic framework nanosheets for the oxygen evolution reaction. Angew. Chem. Int. Ed. **58**, 13565-13572 (2019). https://doi.org/10.1002/anie.201907600.
13. W. Ahn, M. Park, D. Lee, M. Seo, G. Jiang, et al., Hollow multivoid nanocuboids derived from ternary Ni-Co-Fe prussian blue analog for dual-electrocatalysis of oxygen and hydrogen evolution reactions. Adv. Funct. Mater. **28**, 1802129 (2018). https://doi.org/10.1002/adfm.201802129.
14. D. Ding, K. Shen, X. Chen, H. Chen, J. Chen, et al., Multi-level architecture optimization of MOF-templated Co-based nanoparticles embedded in hollow N-doped carbon polyhedra for efficient OER and ORR. ACS Catal. **8**, 7879-7888 (2018). https://doi.org/10.1021/acscatal.8b02504.
15. X. Lu, P. Liao, J. Wang, J. Wu, X. Chen, et al., An alkaline-stable, metal hydroxide mimicking metal-organic framework for efficient electrocatalytic oxygen evolution. J. Am. Chem. Soc. **138** 8336-8339 (2016). https://doi.org/10.1021/jacs.6b03125.
